# Anti-inflammatory microenvironment of esophageal adenocarcinomas negatively impacts survival

**DOI:** 10.1007/s00262-020-02517-8

**Published:** 2020-02-25

**Authors:** Karl-Frederick Karstens, Jan Kempski, Anastasios D. Giannou, Penelope Pelczar, Babett Steglich, Stefan Steurer, Eric Freiwald, Anna Woestemeier, Leonie Konczalla, Michael Tachezy, Matthias Reeh, Maximilian Bockhorn, Daniel Perez, Oliver Mann, Ansgar W. Lohse, Thomas Roesch, Jakob R. Izbicki, Nicola Gagliani, Samuel Huber

**Affiliations:** 1 Department of General, Visceral and Thoracic Surgery, University Medical Center Hamburg-Eppendorf, 20246 Hamburg, Germany; 2 Section of Molecular Immunology and Gastroenterology, Center of Internal Medicine, I. Department of Medicine, University Medical Center Hamburg-Eppendorf, Martinistr. 52, 20246 Hamburg, Germany; 3 Department of Pathology, University Medical Center Hamburg-Eppendorf, Martinistr. 52, 20246 Hamburg, Germany; 4 Department of Medical Biometry and Epidemiology, University Medical Centre Hamburg-Eppendorf, Hamburg, Germany; 5 Department of Interdisciplinary Endoscopy, University Medical Center Hamburg-Eppendorf, 20246 Hamburg, Germany; 6 Immunology and Allergy Unit, Department of Medicine, Karolinska Institute and University Hospital, Solna, Stockholm Sweden

**Keywords:** Esophageal adenocarcinoma, Barrett’s esophagus, Anti-inflammatory environment, Survival

## Abstract

**Objective:**

Reflux promotes esophageal adenocarcinomas (EACs) creating a chronic inflammatory environment. Survival rates are low due to early local recurrences and distant metastasis. Hence, there is a need for new potential treatment options like immunotherapies. However, the inflammatory microenvironment in EACs and its impact on patient outcome remain to be fully understood.

**Methods:**

mRNA expression levels of pro- and anti-inflammatory markers in 39 EAC patients without neoadjuvant radio-chemotherapy were measured. Data were confirmed using flow cytometric analysis of freshly resected surgical specimens. Inflammatory alterations in premalignant lesions of Barrett’s esophagus were analyzed by immunohistochemistry.

**Results:**

Expression levels of *IL22* were reduced in EAC, while expression levels of *FOXP3*, *IL10* and *CTLA4* were increased. Flow cytometry demonstrated a strong infiltration of CD4^+^ T cells with a reduction in CD4^+^ T cells producing IL-22 or IL-17A. We also observed an increase in CD4^+^CD127^low^FOXP3^+^ cells producing IL-10. Accumulation of FOXP3^+^ T cells occurred prior to malignant changes. High expression of *IL10* and low expression of *IL22* in EAC were associated with reduced overall survival. Moreover, increased expression of *IL10*, *CTLA4* and *PD1* in the unaltered esophageal mucosa distant to the EAC was also linked with an unfavorable prognosis.

**Conclusion:**

EAC shows an anti-inflammatory environment, which strongly affects patient survival. The microscopically unaltered peritumoral tissue shows a similar anti-inflammatory pattern indicating an immunological field effect, which might contribute to early local recurrences despite radical resection. These data suggest that using checkpoint inhibitors targeting anti-inflammatory T cells would be a promising therapeutic strategy in EAC.

**Electronic supplementary material:**

The online version of this article (10.1007/s00262-020-02517-8) contains supplementary material, which is available to authorized users.

## Introduction

Patients suffering from gastroesophageal reflux disease (GERD) often present with a condition of chronic inflammation. In some GERD patients, reflux causes a replacement of the squamous epithelium of the esophagus by simple columnar epithelium of intestinal type. This is usually accompanied by goblet cells, which is called Barrett’s esophagus (BE). It has been proposed that these mucosal alterations further progress to dysplastic lesions and ultimately to esophageal adenocarcinomas (EAC) [1]. The incidence of EACs is rising, and despite new multimodal therapies, only 30% of the patients are alive after five years. One reason for poor survival rates is loco-regional recurrences, which have been described in one-third of the patients after curative resection [2–4]. Hence, there is an urgent need for new effective treatments. Immune therapies are currently being investigated, but have not yet entered routine clinical practice [5].

Published data regarding the role of the immune system in the development of EACs have been conflicting [6–9]. Indeed, most studies on esophageal cancer analyze esophageal adenocarcinoma and esophageal squamous cell carcinoma together. However, both entities demonstrate different risk factors, behave in a different biological way and are consequently treated differently [4, 10, 11]. Because of these clear distinctions, we aimed to analyze histopathologically confirmed EACs in association with Barrett’s esophagus. We focused on CD4^+^ T cells which orchestrate the immune system by producing a variety of different cytokines and are known to play a key role in several malignancies. Furthermore, a beneficial effect of inflammation in EACs is supported by recent studies showing that a high infiltration of CD3^+^ and CD8^+^ T cells correlates with a better prognosis [9, 12, 13]. However, a detailed characterization of pro-inflammatory CD4^+^ T cell subsets is missing. Regulatory FOXP3^+^ cells (T_REG_) are one subtype of CD4^+^ T cells. These are usually associated with a poor prognosis in most malignancies [14]. Interestingly, increased numbers of T_REG_ were found in EACs, although their impact on prognosis remains contradictory [8, 13]. One key cytokine product of regulatory T cells is interleukin (IL) 10 [15, 16]. However, the role of IL-10 in tumorigenesis and cancer progression is still controversial and its effect in EAC is unknown [17, 18]. Other studies also indicate that a variety of cytokines play a role in EAC [7–9, 13]. Kavanagh et al. found elevated levels of pro-inflammatory cytokines IL-6, IL-1β, TNF-α and IFN-γ in addition to increased levels of anti-inflammatory IL-10 in the supernatant of EAC biopsies compared to healthy controls, indicating a mixed pro- and anti-inflammatory environment in esophageal adenocarcinomas [7]. In addition, the role of IL-22^+^ and IL-17^+^ T cells in esophageal adenocarcinomas is still unclear. IL-22 and IL-17 are produced by CD4^+^, gamma delta and innate lymphoid cells. Since CD4^+^ T cells are the main source of IL-22 and IL-17, we focused on investigating these cells. Interestingly, several studies have reported a contribution of the latter cytokines to both wound healing and cancer development especially in the context of chronic intestinal inflammation [19–22]. Given that EACs are also usually accompanied by chronic inflammation, we aimed to further characterize the role of IL-22 and IL-17 in this type of tumor.

Immune checkpoint inhibitors, which promote the immune response, have become a promising therapeutic target in cancer. For example, negative costimulatory signals mediated by cell surface proteins such as the programmed cell death protein 1 (PD1) with its programmed cell death ligand 1 or 2 (PDL1 or PDL2) or the cytotoxic T lymphocyte-associated protein 4 (CTLA4) inhibit the activation of T cells and thereby play a central role in anti-tumor immunity in many malignancies [23]. Therefore, the use of immune checkpoint inhibitors is also currently being tested in EACs. Despite these studies, however, the expression profile of these immune checkpoints in EACs is still unclear [24–27]. Before considering the routine use of immune therapies in EACs, it is thus essential to understand the prognostic effect of immune cells, their cytokines and surface receptors. These data could then also form the basis to identify patients, who are most likely to benefit from these immune therapies. This was the other aim of our study.

Based on all of this, we investigated the hypothesis that an anti-inflammatory environment might facilitate tumor development and tumor growth not only in EAC, but also in its non-malignant healthy tissue close to the resection margin leading to early recurrences and poor patient survival. We found an increased infiltration of CD4^+^ T cells with an accumulation of FOXP3^+^ IL-10^+^ secreting T cells in EAC, but a decreased proportion of pro-inflammatory CD4^+^ IL-17^+^ and IL-22^+^ T cells. Interestingly, the non-malignant tissue of EAC patients showed a similar immunological pattern. Accordingly, anti-inflammatory markers were identified as potential prognostic markers indicating worse survival especially in the unaltered peritumoral mucosa independent of other histopathological parameters as shown by multivariate analysis.

## Materials and methods

### Patient samples

Retrospective patient samples were collected from the biobank, and only patients with confirmed Barrett’s esophagus-associated EAC and without neoadjuvant treatment were selected. Samples were obtained from the carcinomas and from peritumoral histomorphologically unaltered esophageal tissue close to the proximal resection margins. For further patient data, see Supplementary Table 1. Esophageal control samples were obtained from healthy volunteers, who underwent routine gastroscopy due to reflux symptoms. The histomorphology of all samples was evaluated using HE staining. Eight samples of the peritumoral tissues were excluded due to infiltration of cancer cells or missing esophageal tissue.

### Isolation of immune cells

Lymphatic cells were isolated from healthy human esophageal mucosa, EAC tissue and its corresponding unaltered tissue close to resection margins. Samples were obtained freshly after esophagectomy from patients diagnosed with EAC. Tissues were washed with PBS. For isolation of intraepithelial lymphocytes (IEL), the esophageal tissue was incubated in HBSS containing 1 mM dithioerythritol (DTE), followed by a dissociation step using 1,3 mM EDTA for 20 min at 37 °C, respectively. To isolate lamina propria lymphocytes (LPL), the tissue was further cut into small pieces and minced with a scalpel. The remaining tissue was incubated for 45 min at 37 °C on a shaking incubator in HBSS (with Ca^2+^ and Mg^2+^) with collagenase (1 mg/ml) and DNase I (10 U/ml), and supernatant was collected. Leukocytes were further enriched by Percoll gradient centrifugation (GE Healthcare). IEL and LPL were collected and pooled.

### Flow cytometry

Human fluorochrome-conjugated antibodies, anti-CD127, anti-CD4, anti-CD3, anti-CD11c, anti-Siglec-8, anti-IL17A, anti-IFNγ, anti-TNF-α, anti-IL22, anti-FOXP3, anti-IL10 all were purchased from BioLegend. Additional anti-human CD3 and anti-human CD4 were used from BD Biosciences. Anti-human IL-22BP antibody (clone 87554) and IgG2B isotype control were obtained from R&D Systems. To identify dead cells, 7-AAD staining (BioLegend) was performed. For extracellular staining, isolated hematopoietic cells from esophageal tissues were incubated for 20 min at 4 °C. Cells were acquired on a LSRII Fortessa flow cytometer (BD). Data were analyzed with FlowJo software (Treestar).

### RNA analysis

Total RNA was extracted from esophageal tissue using TRIzol reagent (Invitrogen). The high-capacity cDNA synthesis Kit (Applied Biosystems) was used for cDNA synthesis. Real-time PCR was performed using the Kapa Probe Fast qPCR Master Mix (Kapa Biosystems) on the StepOnePlus system (Applied Biosystems). For probes used, see Supplementary Table 2. Relative RNA expression was normalized to *HPRT* and calculated using the 2^−∆∆Ct^ method. The mRNA expression levels of *HPRT* across groups were not statistically different (see Fig. S1).

### Immunohistochemistry

Immunohistochemistry was performed on formalin-fixed and paraffin-embedded sections of healthy esophageal tissue, tissue with Barrett’s metaplasia, Barrett’s esophagus containing low-grade (LGD) and high-grade dysplasia (HGD) and early stage (pT1) esophageal adenocarcinomas. An experienced pathologist reviewed the slides for confirmation of morphological changes. DAKO, Monoclonal Mouse Anti-Human CD4, Clone 4B12; eBioscience, Anti-human Foxp3 Purified, Clone 236A/E7 and Abcam, Anti-PD1 antibody (NAT105) ab52587 were used for staining. The sections were counterstained with hematoxylin. CD4, FOXP3 and PD1 presence was determined in a blinded fashion by measuring four fields of visions in the area of interest and calculating the mean.

### Statistical analysis

Statistical analysis was performed with GraphPad Prism^®^ Software (GraphPad Software, San Diego, CA, USA). To compare the groups, the two-sided Mann–Whitney test was used. Significance for Kaplan–Meier curves was calculated by log-rank test. For multivariate survival measurements, statistical analysis was performed using SAS for Windows, version 9.4 (SAS Institute Inc., Cary, NC). Baseline characteristics are given as mean ± standard deviation (SD) and count (proportion) for continuous and categorical factors, respectively. The effect of different variables on time to cancer-related death was investigated using a Cox proportional hazards model. Prior to multivariate survival analysis, the significance was evaluated in a preliminary test using a Cox model. Patients who died within 30 days after surgery were excluded from survival analyses. All analyses were complete case analyses, and therefore, the model was based on 34 patients. Statistical significance was defined as *p* < 0.05.

## Results

### Increased expression of *IL10* and *FOXP3* in EACs

**Fig. 1:**
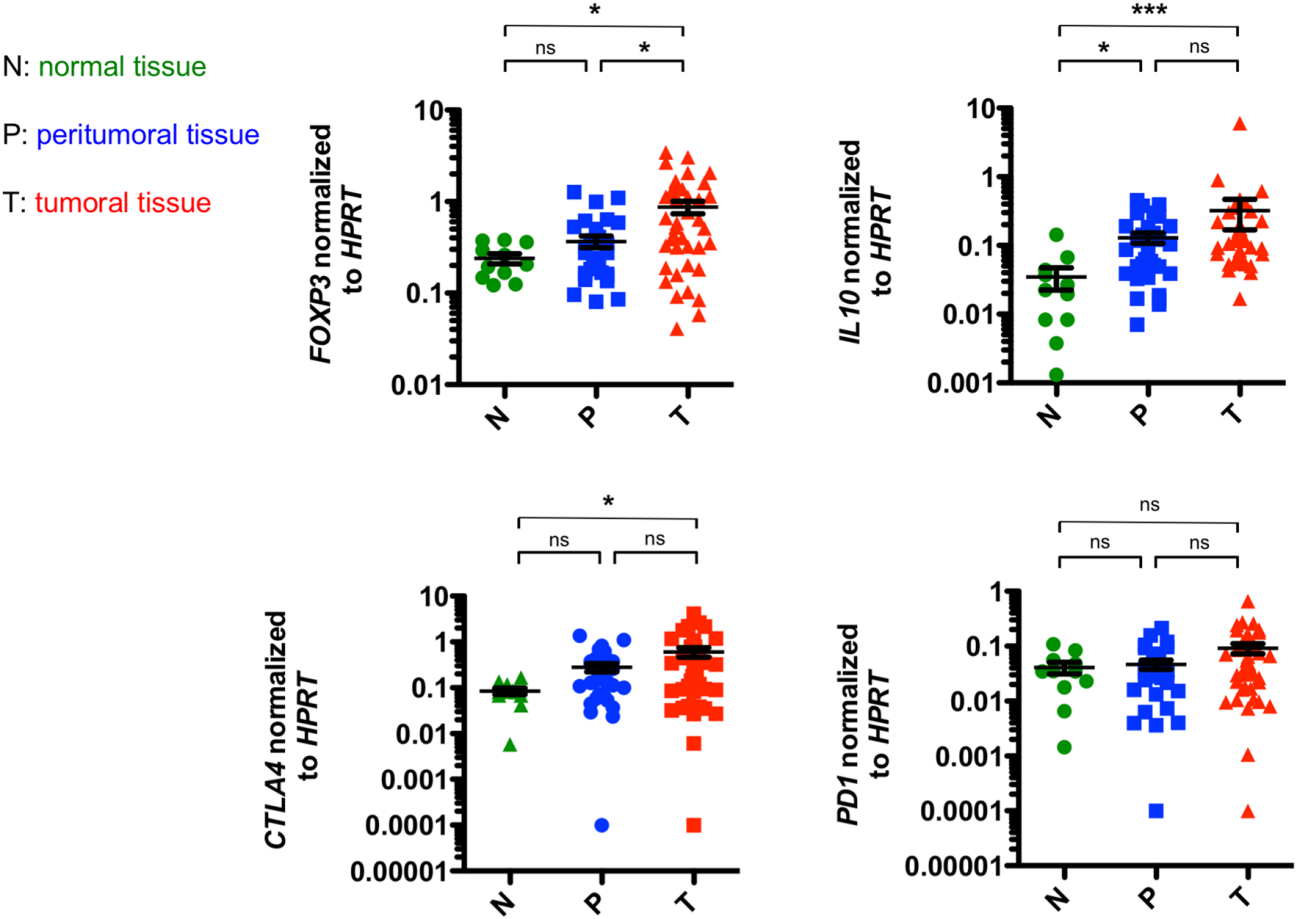
Markers of a regulatory phenotype are increased in esophageal adenocarcinomas and their corresponding unaltered peritumoral tissue. Relative mRNA expression levels of *forkhead box protein 3* (*FOXP3*), *IL10*, *programmed cell death protein 1* (*PD1*) and the *cytotoxic T lymphocyte-associated protein 4* (*CTLA4*) in esophageal tissues from healthy donors (N; *n* = 11), unaltered peritumoral esophageal tissues (P; *n* = 31) and esophageal adenocarcinomas (T; *n* = 39). Data are presented as mean ± SEM. *p < 0.05; **p < 0.01; ***p < 0.001 as assessed by Mann–Whitney *U* test. *p* > 0.05 is considered nonsignificant (ns)

We first aimed to characterize the anti-inflammatory immune response in EAC. We performed a RT-PCR analyzing genes, which are associated with regulatory T cells, such as *FOXP3*, *IL10*, *CTLA4* and *PD1* in EAC, its non-malignant peritumoral tissue and healthy controls. Interestingly, we found that tumoral tissue demonstrated a higher expression of *FOXP3* compared to peritumoral tissue and controls (Fig. 1). In addition, significantly higher levels of *IL10* were found not only in the tumor itself, but also in the peritumoral tissue compared to control samples (Fig. 1). In addition, a trend toward higher levels of *IL10* in locally advanced EACs (T3 and T4 stages) compared to early EACs (T1 and T2 stages) was found (Supplementary Fig. 1). No significant differences in *IL10* expression levels between peritumoral and tumor samples were observed. Interestingly, expression levels of *CTLA4* were higher in tumoral tissue and in peritumoral tissue compared to controls (Fig. 1). However, expression levels failed to reach statistical significance in the latter (*p* = 0.0671). Also, expression levels of *PD1* did not change significantly between the groups (Fig. 1).

### FOXP3^+^ T cells infiltrate EACs

**Fig. 2:**
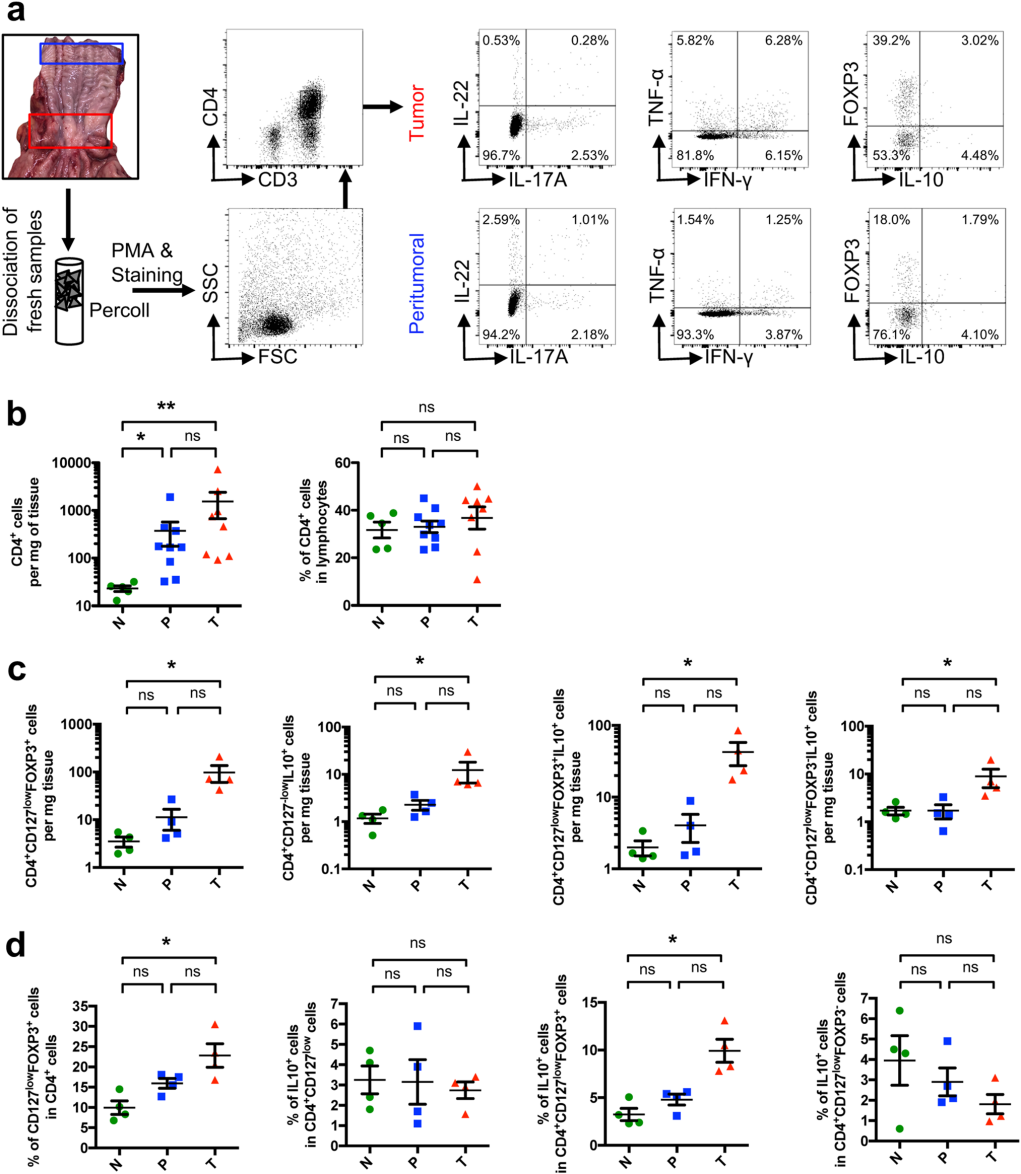
FOXP3^+^ T cells infiltrate EACs and their non-malignant peritumoral tissue. **a** Flowchart of sample localization in the resected specimen, sample processing and staining with representative FACS plots. **b** Total and relative amount of CD4^+^ cells. **c** Amount of CD4^+^CD127^−^FOXP3^+^, CD4^+^CD127^−^IL-10^+^, CD4^+^CD127^−^FOXP3^+^IL-10^+^ and CD4^+^CD127^−^FOXP3^−^IL-10^+^ cells per mg of tissue. **d** Relative amount of cells within their indicated subgroup. All parameters were investigated in esophageal tissues from healthy donors (N; *n* = 4), unaltered peritumoral esophageal tissues (P; *n* = 4) and esophageal adenocarcinomas (T; *n* = 4). Data are presented as mean ± SEM. *p < 0.05; **p < 0.01; ***p < 0.001 as assessed by Mann–Whitney *U* test. *p* > 0.05 is considered nonsignificant (ns)

On the basis of the mRNA expression data suggesting an anti-inflammatory environment, we analyzed freshly resected esophageal specimens (non-malignant peritumoral and tumoral tissue) and healthy esophagus by multi-parameter flow cytometry (Fig. 2a).

We found a significant increase in CD4^+^ T cells per mg tissue in the peritumoral and tumoral samples compared to healthy controls indicating an ongoing immune response (Fig. 2b). The relative frequency of CD4^+^ T cells within the infiltrating lymphocytes remained stable between EAC samples, peritumoral tissues and healthy controls (Fig. 2b).

We measured the frequencies and numbers of CD4^+^CD127^low^FOXP3^+^ T cells and found a significant increase in the relative amount and total numbers of these cells in the tumoral tissue (Fig. 2c, d). Hence, these data indicated a shift within the CD4^+^ T cells toward CD127^low^FOXP3^+^ T cells confirming our results obtained by analyzing the *FOXP3* mRNA expression. Interestingly, microscopically unaltered peritumoral tissue also demonstrated a trend toward an increased infiltration of CD4^+^CD127^low^FOXP3^+^ cells (Fig. 2c, d). Furthermore, we also found an increase in the total number of CD4^+^CD127^low^IL-10^+^ T cells as well as an increase in CD4^+^CD127^low^FOXP3^+^IL-10^+^ and CD4^+^CD127^low^FOXP3^−^IL-10^+^ T cells in the tumor compared to healthy controls (Fig. 2c). In the peritumoral tissue, increased levels of CD4^+^CD127^low^FOXP3^+^IL-10^+^ could be also observed although they did not reach statistical significance compared to healthy controls. The relative amount of CD4^+^CD127^low^FOXP3^+^IL-10^+^ cells in tumoral tissue also increased compared to controls. No significant differences for CD4^+^CD127^low^FOXP3^−^IL-10^+^ cells between all groups were found.

### Characterization of pro-inflammatory cytokine expression patterns in EACs

To further sustain the hypothesis that EAC has an anti-inflammatory environment, we next assessed the pro-inflammatory immune response. We specifically wanted to study whether healthy esophageal tissue and malignant EAC tissue show distinct pro-inflammatory profiles. In addition, we aimed to investigate whether microscopically verified unaltered esophageal tissue of EAC patients demonstrates a different cytokine pattern compared to carcinomas or control samples. To this end, we first analyzed the mRNA levels of inflammatory cytokines and their receptors (*IL22*, *IL22BP*, *IL22RA1*, *IL17A*, *IFNγ, TNFα*) on resected specimens.

**Fig. 3:**
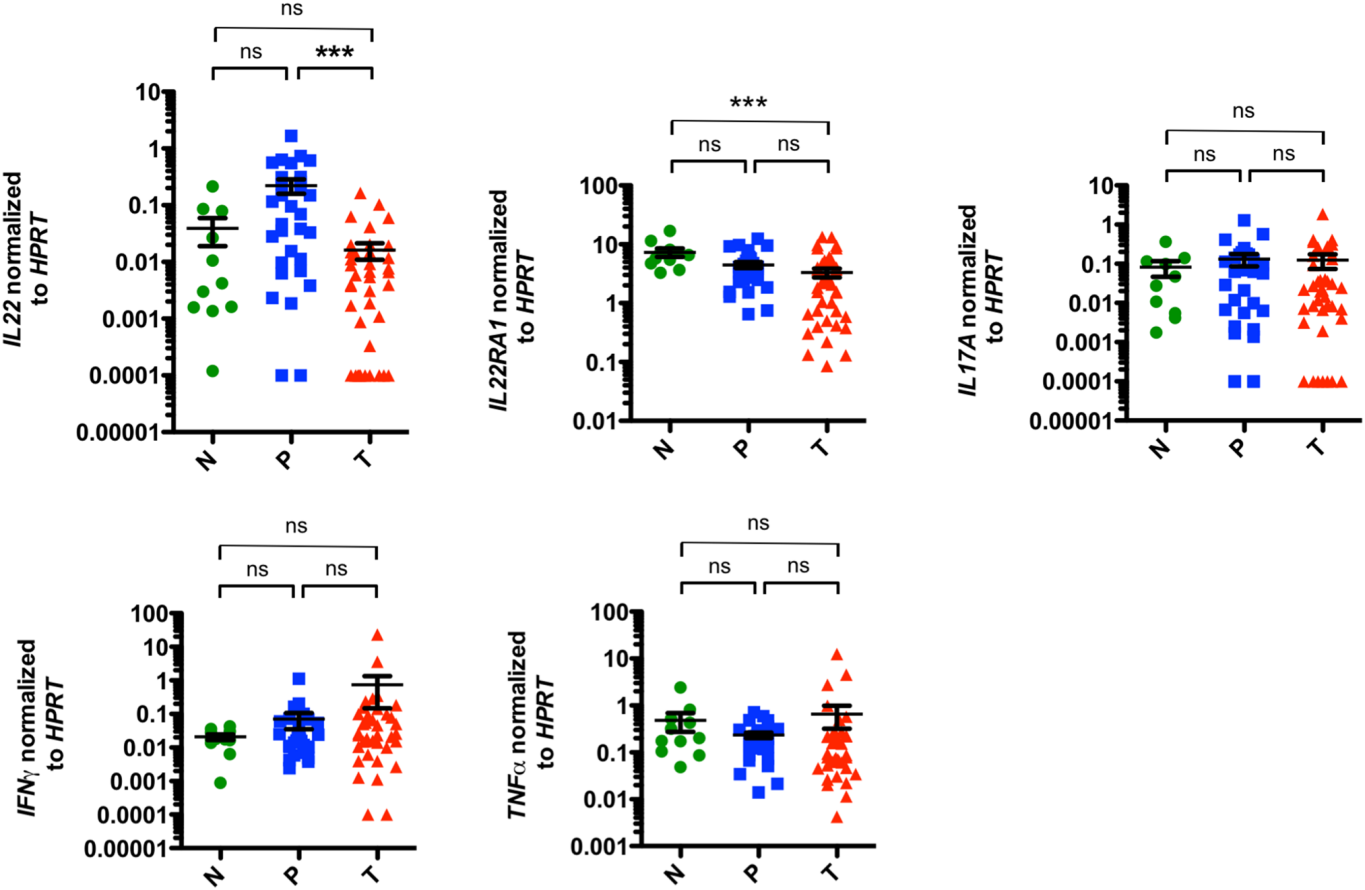
*IL22* mRNA expression levels are reduced in esophageal adenocarcinomas. Relative mRNA expression levels of *IL22*, *IL22RA1*, *IL17A*, *IFNγ* and *TNFα* in esophageal tissues from healthy donors (N; *n* = 11), unaltered peritumoral esophageal tissues (P; *n* = 31) and esophageal adenocarcinomas (T; *n* = 39). Data are presented as mean ± SEM. *p < 0.05; **p < 0.01; ***p < 0.001 as assessed by Mann–Whitney *U* test. *p* > 0.05 is considered nonsignificant (ns)

We observed a decrease in *IL22* expression in tumoral tissue compared to peritumoral samples and healthy controls (Fig. 3). However, it failed to reach statistical significance in the latter. No significant differences between peritumoral and control samples were found. When we analyzed *IL22BP*, the soluble receptor of IL-22 also known as IL-22RA2, we observed no differences in the expression levels between all investigated groups (Fig. S3a). Interestingly, expression levels of the IL-22 receptor 1 (*IL22RA1*) demonstrated to be significantly reduced in the tumors compared to healthy controls (Fig. 3). When we analyzed *IL17A* expression levels in our cohort, no significant differences between all groups were found (Fig. 3). However, significant lower IL17A levels were found in locally advanced EACs (T3 and T4 stages) compared to early tumors (T1 and T2 stages) (Fig. S2). Expression levels of *IFNγ* increased from healthy tissue over peritumoral tissue to EACs, but no statistical significance could be reached (Fig. 3). Finally, we analyzed the mRNA expression of *TNFα*. However, no significant changes in *TNFα* expression levels were detected in the investigated groups (Fig. 3).

### A relative reduction in effector T cells is found in EACs and its non-malignant peritumoral tissue

**Fig. 4:**
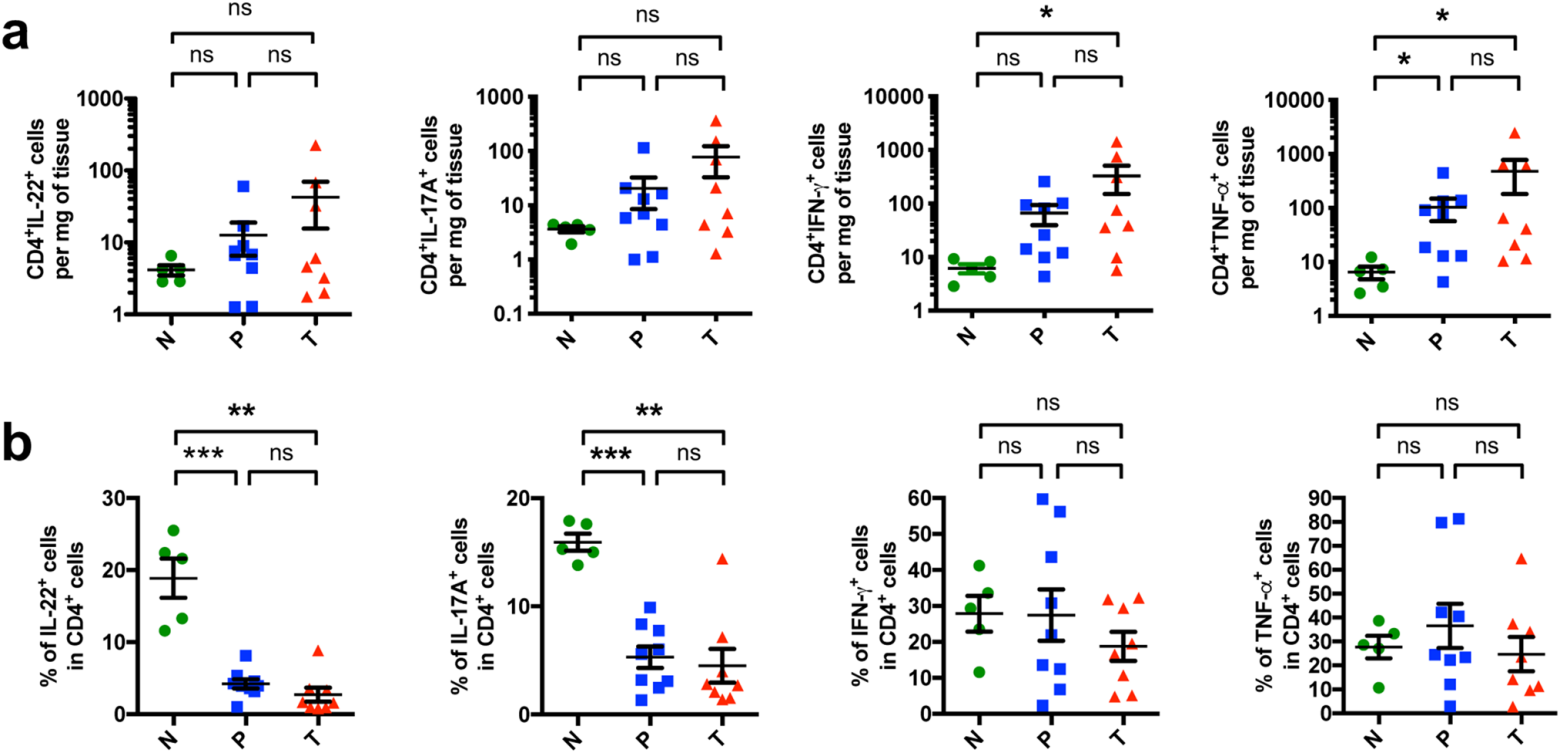
CD4^+^ T cells with a relative reduction in effector T cells infiltrate EACs and their non-malignant peritumoral tissues. **a** Amount of CD4^+^IL-22^+^, CD4^+^IL-17A^+^, CD4^+^IFN-γ^+^ and CD4^+^TNF-α^+^ cells per mg of tissue. **b** Relative amount of IL-22^+^, IL-17A^+^, IFN-γ^+^ and TNF-α^+^ cells within the CD4^+^ T cell population. All parameters were investigated in esophageal tissues from healthy donors (N; *n* = 5), peritumoral esophageal tissues (P; *n* = 9) and esophageal adenocarcinomas (T; *n* = 8). Data are presented as mean ± SEM. *p < 0.05; **p < 0.01; ***p < 0.001 as assessed by Mann–Whitney *U* test. *p* > 0.05 is considered nonsignificant (ns)

To further characterize the pro-inflammatory immune response, we performed multi-parameter flow cytometry on freshly resected surgical samples. We found by trend a higher total number of IL-22^+^ and IL-17A^+^ CD4^+^ T cells in the tumors compared to healthy controls (Fig. 4a). Interestingly, when analyzing the relative proportion of IL-22^+^ producing cells within the CD4^+^ T cells, a significant decrease in the tumors and peritumoral tissues was observed (Fig. 4b). In addition, the relative proportion of IL-17A^+^ cells within the CD4^+^ T cells decreased not only in the tumors but also in the peritumoral tissues compared to healthy controls (Fig. 4b).

When looking into the soluble inhibitory receptor of IL-22, a decrease in the amount of IL-22BP producing CD4^+^ T cells in the tumor tissue was found (Supplementary Fig. 2b). Since the main sources of IL-22BP in the colon were CD11c^+^ cells, we particularly checked the IL-22BP expression in this subgroup. Also, CD11c^+^ cells showed a reduced IL-22BP production in EAC compared to healthy controls (Fig. S3c). We then tested the frequency and number of IFN-γ^+^ and TNF-α^+^ CD4^+^ T cells. We observed that an increased amount of these cells was present in the tumor compared to healthy tissue (Fig. 4a). In addition, the amount of CD4^+^TNF-α^+^ T cells in peritumoral tissue was significantly higher than in controls (Fig. 4a). However, no changes were seen in the proportion of infiltrating IFN-γ^+^ and TNF-α^+^ cells within the CD4^+^ T cells (Fig. 4b).

### Increased infiltration of CD4^+^, FOXP3^+^ and PD1^+^ T cells precedes malignant mucosal changes

**Fig. 5:**
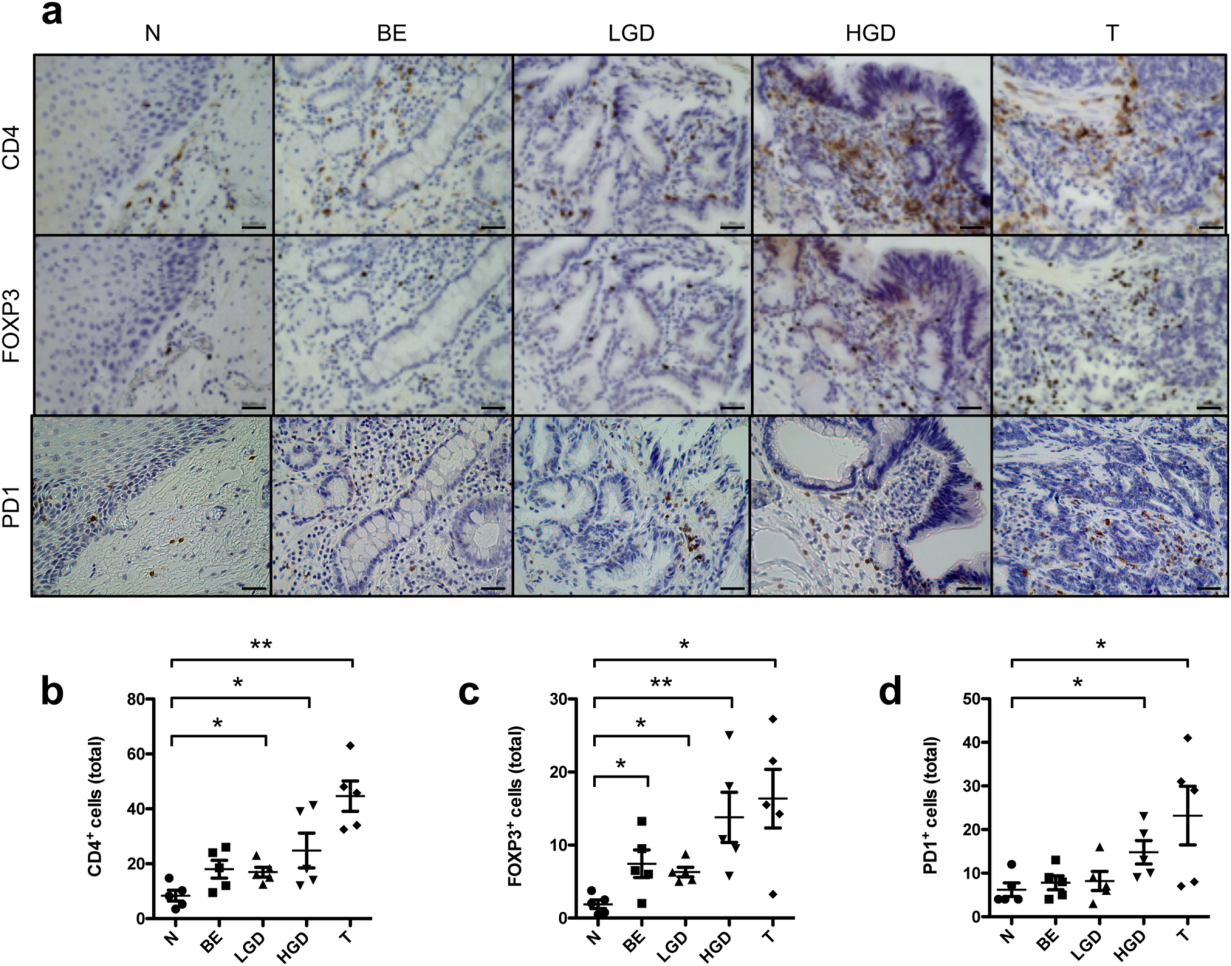
Increased infiltration of CD4^+^ cells and FOXP3^+^ T cells precede malignant mucosal changes. **a** Representative immunohistological pictures of normal tissue (N), Barrett’s metaplasia (BE), BE with low-grade dysplasia (LGD), BE with high-grade dysplasia (HGD) and early stage esophageal cancer (T) with staining for CD4, FOXP3 and PD1. Bar indicates 20 µm. **b** Cumulative results for the total amount of CD4^+^ cells in N, BE, LGD, HGD and T. **c** Cumulative results for the total amount of FOXP3^+^ cells in N, BE, LGD, HGD and T. **d** Cumulative results for the total amount of PD1^+^ cells in N, BE, LGD, HGD and T. Only significant parameters are marked for better visualization. Data are presented as mean ± SEM. *p < 0.05; **p < 0.01; ***p < 0.001 as assessed by Mann–Whitney *U* test. *p* > 0.05 is considered nonsignificant

To further confirm the above-mentioned data indicating a shift from pro- to anti-inflammatory T cells in EAC, we analyzed the protein expression levels of CD4, FOXP3 and PD1 in paraffin-embedded specimens of healthy esophagus, Barrett’s esophagus, mucosa with low-grade and high-grade dysplasia and early stage (pT1) EACs (Fig. 5a). We found a significant increased infiltration of CD4^+^ T cells in low- and high-grade dysplasia and in EACs compared to normal esophageal mucosa (Fig. 5b). Also, an increase in FOXP3^+^ T cells was found in mucosal alterations preceding EACs and in EACs themselves (Fig. 5c). In addition, PD1^+^ T cells were found more frequently in mucosal samples with high-grade lesions and early stage EACs (Fig. 5d). No differences between the other groups were observed.

### Increased expression levels of *IL10*, *CTLA4* and *PD1* are associated with poor overall survival

**Fig. 6:**
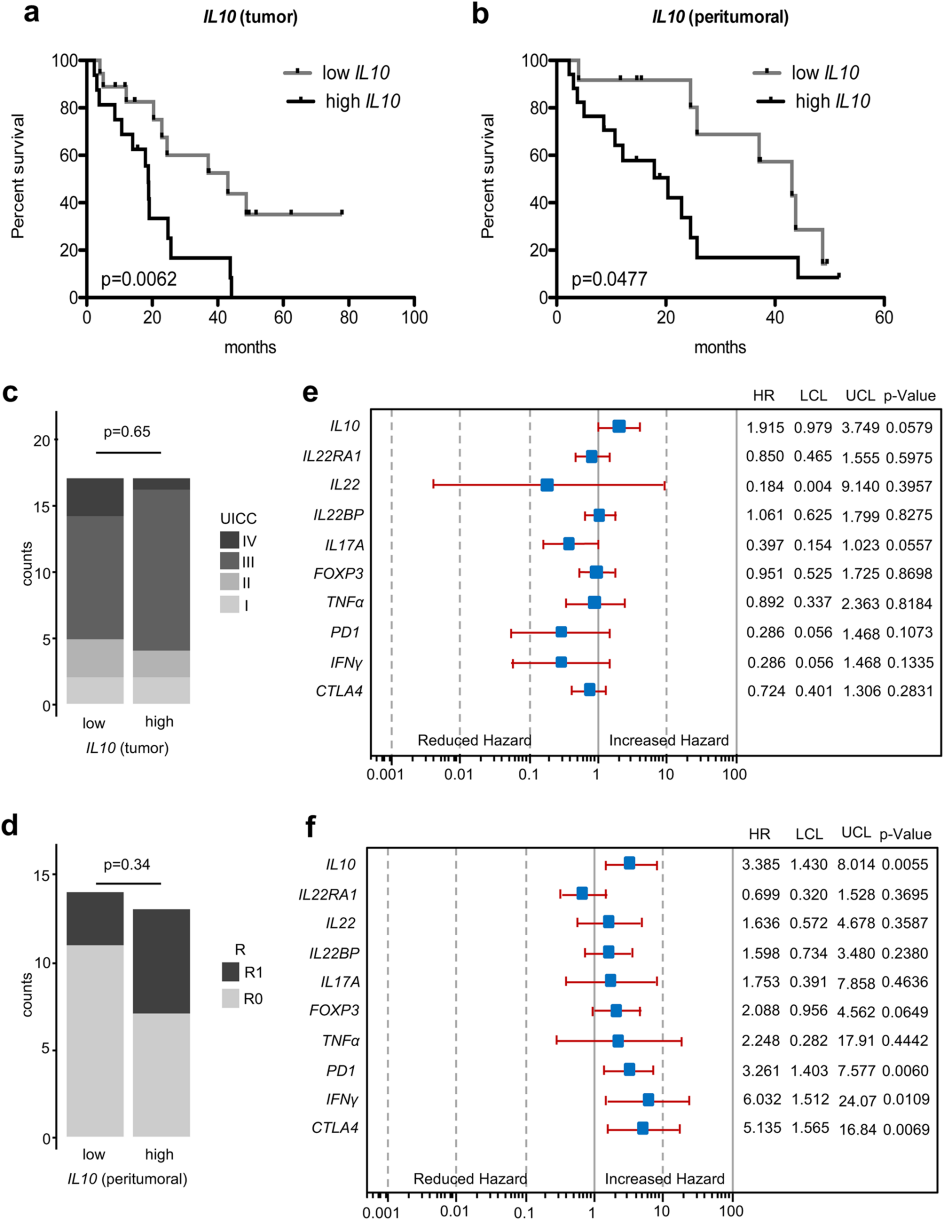
Increased expression levels of anti-inflammatory markers, especially in the unaltered mucosa of EAC patients, are associated with poor overall survival. **a**, **c** Kaplan–Meier curve comparing the survival of patients with high and low relative *IL10* mRNA expression in EAC tissue (median as cutoff) with the distribution of UICC stages. **b**, **d** Kaplan–Meier curve comparing the survival of patients with high and low relative *IL10* mRNA expression in peritumoral tissue (median as cutoff) with the distribution of the resection (R) status. **e**, **f** Multivariate analysis showing the effect of relative cytokine mRNA expression levels on patient survival in EAC and peritumoral tissue. Patients that did not survive the first 30 days after surgery were excluded from all survival analysis

To further address the clinical implications of our findings, we investigated whether distinct cytokines and co-inhibitory receptor expression correlate with patients’ prognosis. When correlating mRNA expression with overall survival rates in univariate analysis, high *IL10* levels in both tumor and peritumoral samples were significantly associated with worse patient prognosis (Fig. 6a, b). In peritumoral samples, high expression levels of *PD1* were also associated with worse survival (Fig. S4). In addition, reduced expression levels of *IL22* in tumoral tissues were associated with poor patient survival (Fig. S5). As expected, UICC stages were also associated with reduced survival (Fig. S6). Of note, no significant differences in the distribution pattern of UICC stage or resection status were found in the analyzed tumoral or peritumoral groups, respectively (Fig. 6c, d).

We then tested whether the investigated cytokines influenced survival independently of clinical and histopathological parameters by multivariate analysis. A backward Cox analysis was performed to exclude parameters without impact on survival. In this analysis, only sex, age at time of surgery, T-, N- and G-status and reflux yielded a significant impact on survival. Hence, these parameters were tested against different variables of mRNA expression levels. In tumor tissue, *IL10* expression levels missed a significant correlation with patient survival by a small margin (Fig. 6e). In contrast, histologically unaltered peritumoral samples with high *IL10* expression levels yielded a strong correlation with poor patient survival (Fig. 6f). Interestingly, in peritumoral tissues, increased *CTLA4*, *PD1* and *IFNγ* expression levels were also significantly associated with worse overall survival (Fig. 6f). When taking the surgical resection status (R status), which indicates tumor-free margins into multivariate analysis, all previously investigated parameters remained significant. In addition, the influence of *IL10* in EACs on overall survival turned to be significant. Also, *FOXP3* levels in the peritumoral tissue now demonstrated a significant correlation (Fig. S7).

## Discussion

EAC is a disease with poor survival rates that still lacks an effective systemic treatment option. This is one of the few studies, which focused on patients whose immune response was not altered because of chemotherapy. This is because we obtained samples from a unique cohort of patients that did not receive any neoadjuvant therapy. Pro- and anti-inflammatory cytokines have both been described in EACs. However, their effect on patient survival had not yet been investigated. Moreover, we analyzed the immune system also in the histologically unaltered esophageal tissue close to the oral resection margin.

Our data indicate a strong influence of IL-10 on patient survival in EACs irrespective of other clinical or histopathological parameters. In gastric cancer patients, increased mRNA levels of *IL10* were also associated with worse survival in multivariate analysis [18]. Of note, we found that in the unaltered peritumoral tissue, *IL10* was a stronger predictor of survival compared to EACs. In line with the latter finding, Fitzgerald et al. observed increased levels of IL-10 in the distal parts of BE. In these parts of BE, the least inflammation was reported and four of the six EACs occurred later on [28]. In addition, an in vitro analysis detected increased levels of IL-10 in the supernatant of Barrett’s mucosa and EACs compared to healthy controls [7]. These data underline the immunosuppressive function of IL-10 and support our findings regarding the prognostic relevance of IL-10 even if no malignancy has occurred.

Supporting the hypothesis of a predominantly anti-inflammatory environment in EACs, we also found more CD4^+^CD127^low^FOXP3^+^ cells in EACs and in its unaltered peritumoral tissue and less effector T cells. We did not only identify a significant increase in FOXP3^+^ cells in late stage EACs, but also in early stage EACs and prior to malignant mucosal changes. Somja et al. also reported a significant increase in FOXP3^+^ cells during the dysplasia–carcinoma frequency in Barrett’s esophagus [29]. Hence, CD4^+^CD127^low^FOXP3^+^ cells, which are usually classified as regulatory T cells (T_REG_), might contribute to tumor development by suppressing pro-inflammatory cytokines. In line with the anti-inflammatory role of T_REG_, a reduced number of CD4^+^ CD45RO^+^ memory T cells were observed in EACs [7]. In addition, Rauser et al. showed a positive correlation between high density of CD45RO^+^ T cells and disease-free survival in stage I–IIa patients [12]. Stein et al. also demonstrated that a high amount of intratumoral tissue-infiltrating lymphocytes (TIL) and a high grade of total inflammation were beneficial for the prognostic value in EACs [13]. However, Zingg et al. failed to show a prognostic effect on the number of TILs [8]. When specifically looking at FOXP3^+^ cells, only Stein et al. were able to find a positive correlation between high infiltration of FOXP3^+^ T cells and patient survival. In our cohort, elevated *FOXP3* expression levels correlated with worse patient survival in the peritumoral group after R status was taken into account. Taken together, our findings suggest that IL-10 and FOXP3^+^ cells are an evidence of an anti-inflammatory response to a chronic inflammation, which can be detected in other cytokines such as IFN-γ and TNF-α. Hence, IL-10 and FOXP3^+^ T cells might serve as a potential prognostic biomarker in BE and EAC. However, further studies are warranted to test this hypothesis.

Furthermore, we found an increase in *CTLA4* expression in peritumoral as well as in tumoral samples of EAC patients. This datum sustains our finding that an anti-inflammatory environment characterizes EACs. Unexpectedly, in multivariate analysis, *CTLA4* expression only correlated with patient survival in the unaltered peritumoral group. In patients with ESCC, increased CTLA4 levels have been implicated to be associated with shorter overall survival in multivariate analysis [30]. Hence, patients with increased *CTLA4* expression in EACs might benefit from a CTLA4 blockade.

Another important receptor for suppressing T cell activation is PD1. The PD1/PD-L pathway plays an important role in the regulation of the immune cells against tumor cells [23]. Derks et al. reported PD1^+^ infiltrating lymphocytes in 59.8% of the investigated EACs. They were also able to demonstrate a correlation with T stage and grading [25]. In our study, relative *PD1* expression levels did not significantly differ between EAC and controls. However, we found an increase in PD1^+^ T cells in high-grade dysplasia and in EACs themselves. In addition, increased *PD1* levels demonstrated to be worse for overall survival in the peritumoral group. Our findings emphasize PD1 as a potential target in EACs for immune therapies. Accordingly, in one of the first studies testing the treatment of PD1-blockade as third line therapy in advanced gastric or gastroesophageal junction cancer, a significant benefit for PD1-blockade was found [31]. However, it is becoming evident that not only the presence of PD1, but rather the local and systemic composition of inflammatory cells reacting to the PD1/PD-L pathway is important for the response to the anticancer immune therapy [32, 33].

In Barrett’s esophagus, increased expression levels of IFN-γ and TNF-α have been reported and TNF-α seems to increase along the metaplasia–dysplasia–carcinoma sequence [6, 34]. However, we did not find any significant differences between RNA expression levels of IFN-γ and TNF-α, nor did we find any significant changes in the relative amount of infiltrating CD4^+^ IFN-γ^+^ and CD4^+^ TNF-α^+^ T cells between all groups. This confirms the findings of a recent study, in which no significant differences in the relative amounts of CD4^+^ IFN-γ^+^ and CD4^+^ TNF-α^+^ cells were reported in EACs [7]. Notably, we found that *IFNγ* expression levels in peritumoral tissues showed to be worse for patients’ prognosis. Usually, *IFNγ* expression is associated with a superior outcome in most cancer entities, such as colorectal cancer [35]. The underlying reason is currently unknown. However, our results could be explained by findings of other studies, which have reported an induction of PD-L1 expression by IFN-γ leading to a potent immune escape in certain malign cells [36, 37], thus adding to an anti-inflammatory environment.

To further characterize the pro-inflammatory environment, we investigated the IL-22/IL-22BP axis and IL-17A^+^ cells in EACs. Several immune cells, including innate lymphoid cells and CD4^+^ T cells, produce IL-22. IL-17^+^ and IL-22^+^ which are involved in both wound healing and tumor development through activation of STAT3 [20, 21]. In addition, it has been shown that IL-22 induces DOT1L in colon epithelial cells leading to the expression of *Nanog*, *Sox2* and *Pou5F1*. The latter genes cause stem cell-like characteristics in colon cells, which might contribute to tumor growth [38]. In contrast, IL-22 also bears anti-tumor effects since blocking of IL-22 in early tumor initiation stages during DSS colitis leads to a reduced tumor burden. However, when blocking IL-22 in later stages, a pro-tumorigenic effect was observed [19]. These data are in line with a recent study indicating that IL-22 is protective against early genotoxic events in the colon [39]. Hence, multiple factors determine the biological function of IL-22. In addition, IL-22 binding protein has been described as a inhibitory soluble receptor of IL-22, which is expressed by a subset of dendritic cells [40, 41]. The balance of the IL-22-IL-22BP axis seems to be critical for tumorigenesis in the colon [19]. IL-17^+^ T helper cells are thought to be involved in immunoregulation and primarily secrete IL-17A, IL-17F and IL-22. However, their precise role is still under debate [42]. In squamous cell carcinomas of the esophagus (ESCC), IL-17^+^ infiltrating cells seem to be anti-tumorigenic and were reported to positively correlate with a good patients’ prognosis [43, 44]. In the progression from healthy tissue toward EACs, increased IL-17^+^ cells have been found [45]. Here, we describe that IL-22^+^ and IL-17^+^ effector T cells were reduced in EAC and low *IL22* expression was associated with worse survival in univariate analysis. The reduction in IL-17A^+^ cells in EACs is in line with a finding from Kavanagh et al. [7] who also reported a reduced proportion of CD4^+^ IL17^+^ cells in EAC compared to normal controls. The findings of reduced effector T cells emphasize the dominance of an anti-inflammatory environment in EACs. However, IL-22 has also been reported to function in cell regeneration on mucosal barriers. We observed elevated *IL22* expression and IL-22^+^ cells in the peritumoral tissue compared to tumor tissue. Hence, one might speculate that in the unaltered peritumoral tissues, cytokine-mediated repair mechanisms of the mucosa are still intact, while in EACs, reduced IL-22 leads to impaired cytokine-mediated cell regeneration. We also found a reduced *IL22RA1* expression in tumor tissue. Since IL22RA1 is especially expressed on barrier surfaces, low expression levels could be due to replacement of the physiological esophageal mucosa by cancer cells. In addition, Gronke et al. very recently showed that stem cells deprived of IL-22 and exposed to carcinogens were able to escape apoptosis, had more mutations and were more likely to give rise to colon cancer [39]. Thus, these data indicate a possible imbalance of IL-22 signaling axis in EACs, which might accelerate tumorigenesis and impair wound healing.

We found that the anti-inflammatory environment in the microscopically unaltered peritumoral tissue had a stronger impact on patient survival compared to tumoral tissue. One explanation could be the surgery itself since all cancer tissue is resected with safety margins to achieve an oncologic resection. However, the remaining tissue close to resection margins might already contain an anti-inflammatory environment and thus be prone to early recurrences. Hence, it might strongly influence overall survival. In addition, the similarity of the immunological profile in the unaltered peritumoral tissue compared to the malignant tissue was surprising. These data indicate a potential immunological field effect around EACs, which might also contribute to tumor progression in already existent EACs and might lead to tumor development or local recurrence despite radical resection.

In conclusion, we show that CD4^+^CD127^low^FOXP3^+^ T cells infiltrate EACs, while CD4^+^ IL-22^+^ and IL-17^+^ effector T cells are reduced. FOXP3^+^ T cells can be found even prior to malignant mucosal changes. This might indicate a shift from a chronic pro-inflammatory environment toward an anti-inflammatory environment during EAC development. We for the first time describe an anti-inflammatory field effect in Barrett’s esophagus-associated cancers that might contribute to early local recurrences in the unaltered mucosa. Further, an association of *CTLA4* and *PD1* expression with reduced survival was found. In addition, we demonstrated a strong IL-10 expression in EACs, which negatively impacts survival independently of other clinical or histopathological parameters. Thus, our data indicate that *IL10, CTLA4* and *PD1* expression could serve as a biomarker to identify patients, who might benefit from closer surveillance and immunotherapy using checkpoint inhibitors.

## Electronic supplementary material

Below is the link to the electronic supplementary material.

Supplementary material 1 (PDF 942 kb)

## Abbreviations

BE: Barrett’s esophagus
BMI: Body mass Index
CTLA4: Cytotoxic T lymphocyte-associated protein 4
DOT1L: Disruptor of telomeric silencing 1-like
DSS: Dextran sulfate sodium
DTE: Dithioerythritol
EAC: Esophageal adenocarcinoma
G status: Grading of tumor cells
GERD: Gastroesophageal reflux disease
IEL: Intraepithelial lymphocytes
LPL: Lamina propria lymphocytes
M status: Distant metastasis
N status: Tumor involvement in nearby lymph nodes
STAT3: Signal transducer and activator of transcription 3
T status: Size and depth of tumor invasion
UICC: Union for International Cancer Control (comprising TNM status)
