# Supplementary material for: Anti-inflammatory microenvironment of esophageal adenocarcinomas negatively impacts survival
Source: Cancer Immunol Immunother. 2020 Feb 25;69(6):1043–56. doi: 10.1007/s00262-020-02517-8 (PMC7230052; doi:10.1007/s00262-020-02517-8)
Supplement: Supplementary file 1 — Supplementary material 1 (PDF 942 kb) [file 262_2020_2517_MOESM1_ESM.pdf]

SUPPLEMENTARY MATERIAL

Figure S1: *HPRT* levels among the investigated groups

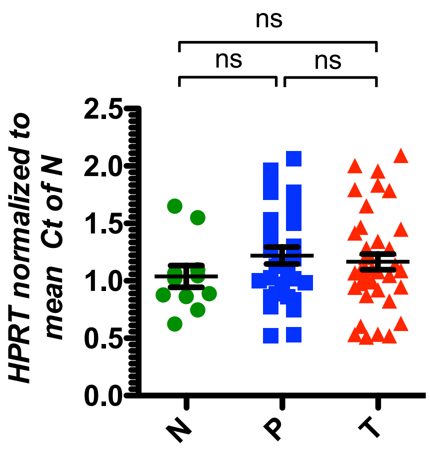

Relative mRNA expression levels of *HPRT* in esophageal tissues from healthy donors (N; n=11), esophageal adenocarcinomas (T; n=39) and unaltered peritumoral esophageal tissues (P; n=31). The mean Ct of the control group (N) was determined and used as reference for the $2^{-\Delta\Delta Ct}$ method to calculate values for each group. p>0.05 is considered non-significant (ns).

Figure S2: Increased *IL10* and decreased *IL17A* expression are associated with advanced local tumor infiltration and grading.

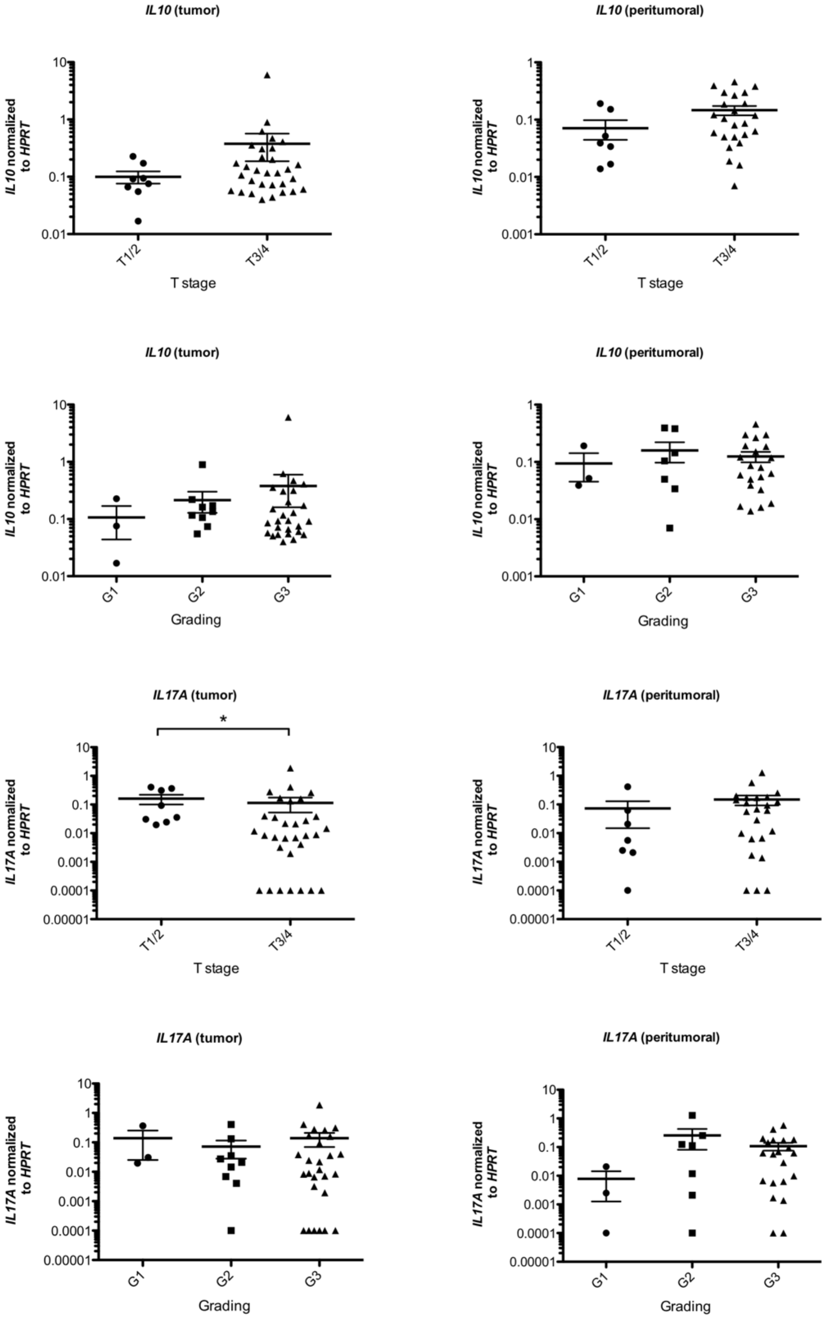

Only significant parameters are marked for better visualization. Data are presented as mean ± SEM. * = $p<0.05$; ** = $p<0.01$; *** = $p<0.001$ as assessed by Mann-Whitney-U test. $p>0.05$ is considered non-significant.

Figure S3: *IL22BP* levels did not change significantly between the investigated samples.

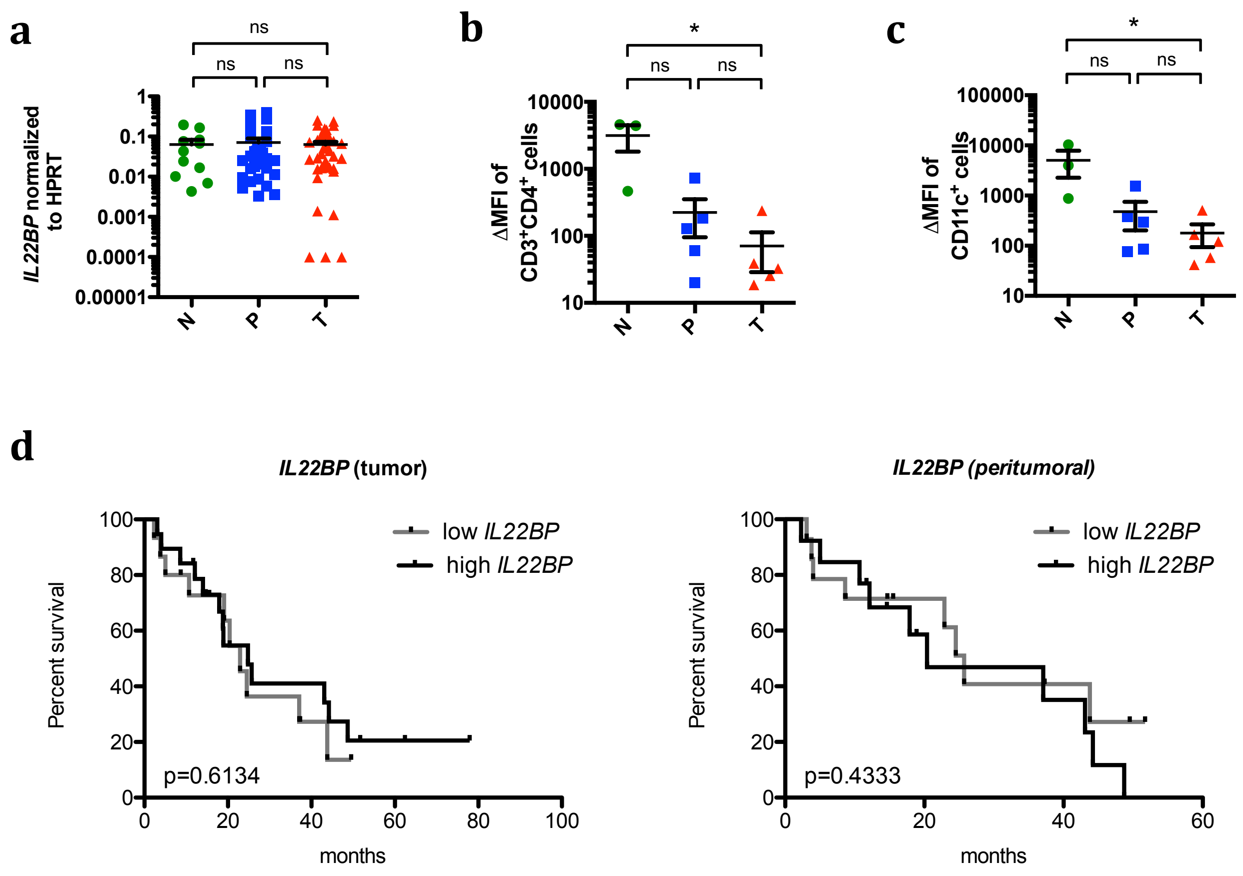

a) Relative mRNA expression levels of *IL22BP* in esophageal tissues from healthy donors (N; n=11), unaltered peritumoral esophageal tissues (P; n=31) and esophageal adenocarcinomas (T; n=39). **b & c)** ΔMFI in $CD3^{+}CD4^{+}$ and $CD11c^{+}$ cells in esophageal tissues from healthy donors (N; n=3), unaltered peritumoral esophageal tissues (P; n=5) and esophageal adenocarcinomas (T; n=5), respectively. **d)** Kaplan-Meier curve comparing the survival of patients with high and low relative *IL22BP* mRNA expression in EAC and peritumoral tissue (median as cutoff). Data are presented as mean ± SEM. * = p<0.05; ** = p<0.01; *** = p<0.001 as assessed by Mann-Whitney-U test. p>0.05 is considered non-significant (ns).

Figure S4: Kaplan-Meier curves of relative mRNA expression levels for *FOXP3*, *CTLA4* and *PD1.*

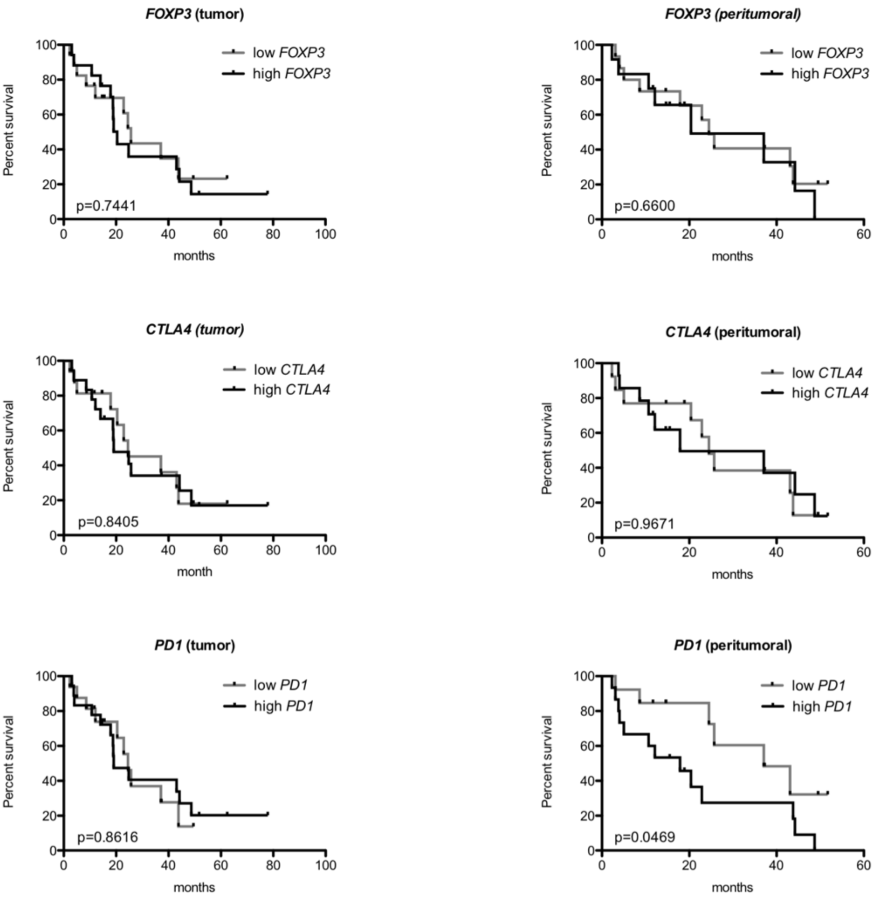

Median was used as cutoff. Significance was analyzed by log rank test.

Figure S5: Kaplan-Meier curves of relative mRNA expression levels for *IL22*, *IL22RA1*, *IL17A*, *IFNγ* and *TNFα*.

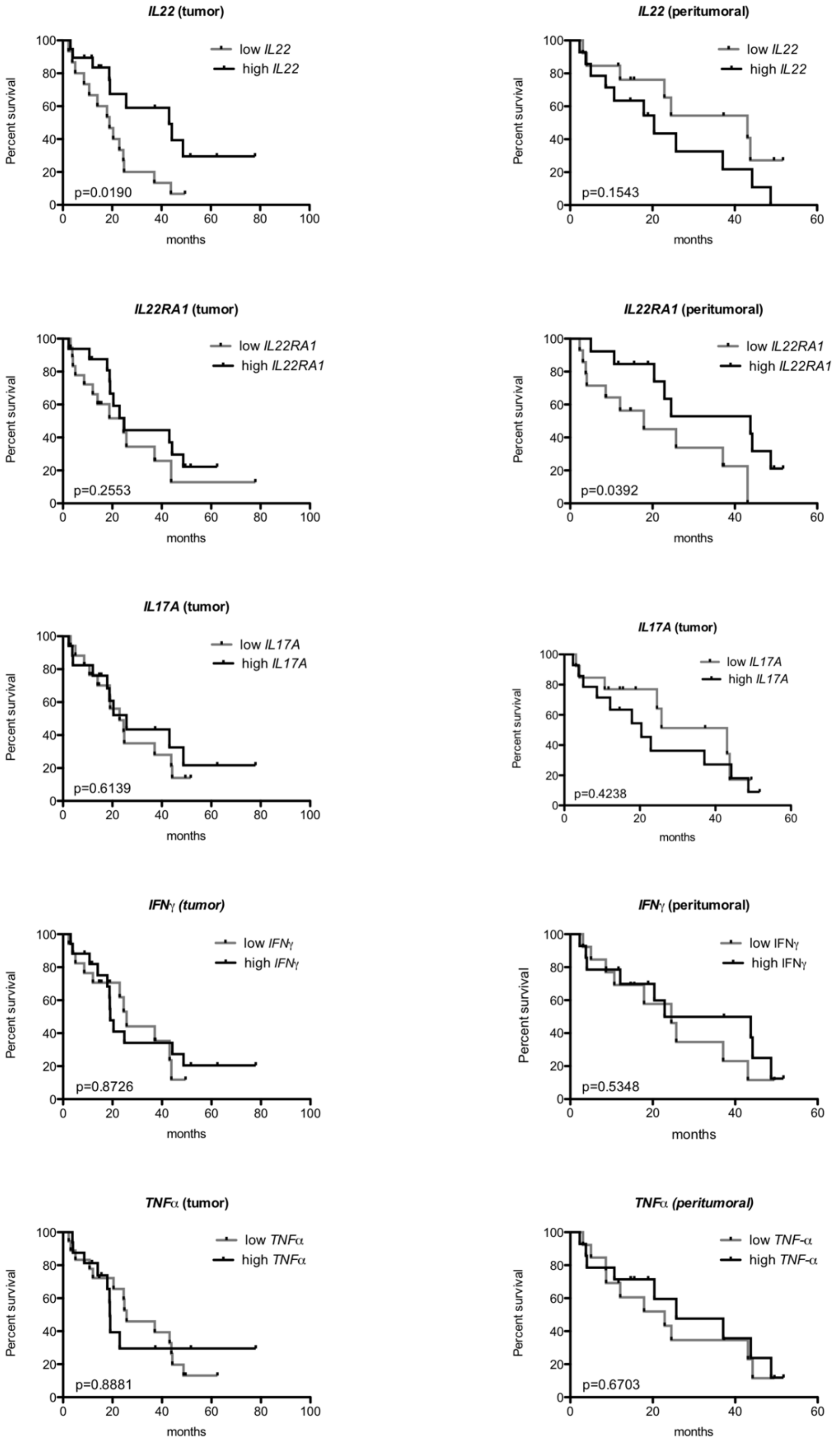

Median was used as cutoff. Significance was analyzed by log rank test.

Figure S6: Kaplan-Meier curves of UICC stages.

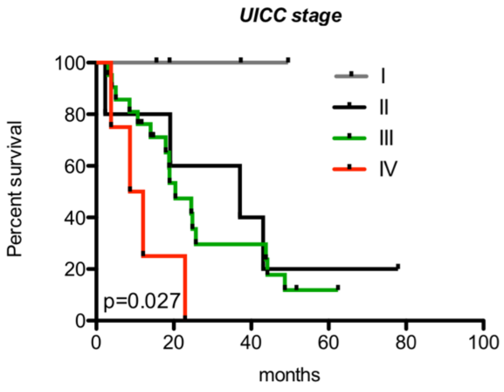

Significance was analyzed by log rank test.

Figure S7: Cox-regression analysis for the investigated cytokines with R status.

a

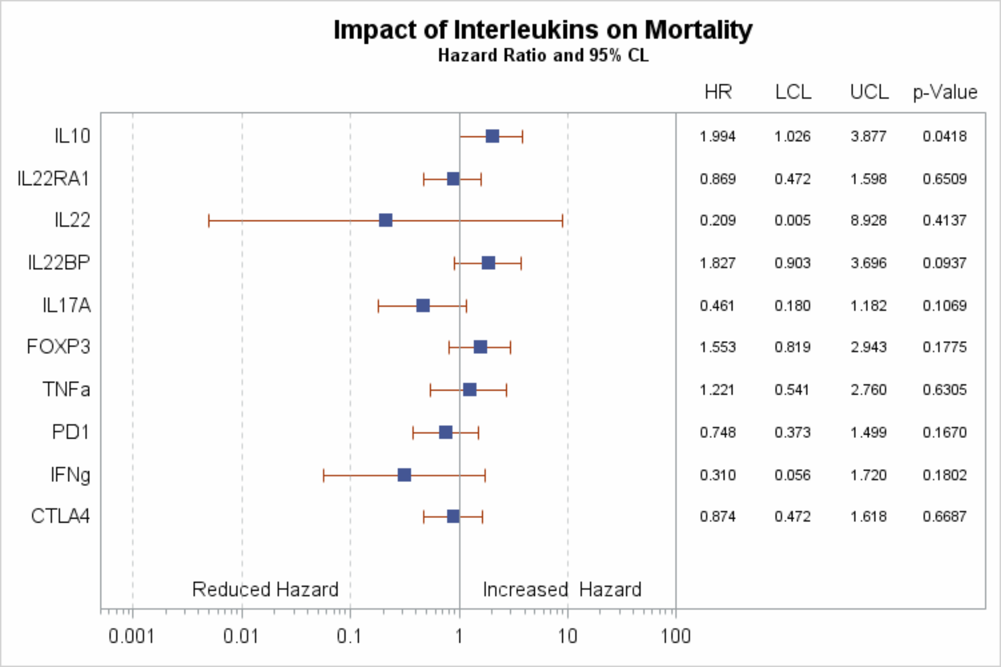

b

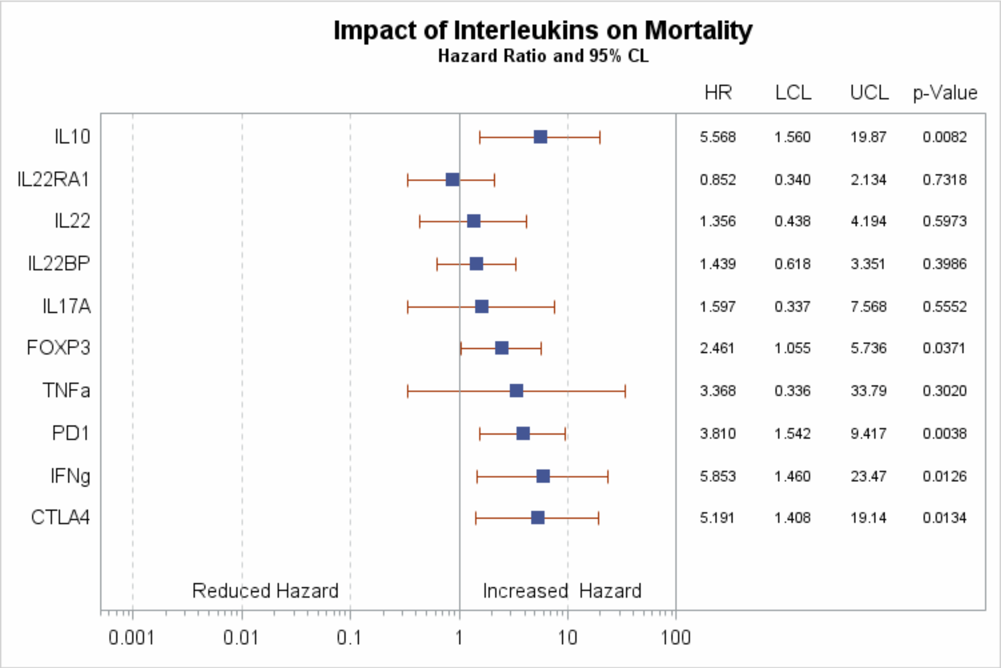

a) Cox-regression analysis for cytokines in tumor tissue. **b)** Cox-regression analysis for cytokines in peritumoral tissue. HR: hazard ratio; LCL: lower confidence level; UCL: upper confidence level.

Table S1: Clinical data of patients enrolled in this study.

		all patients (n=39)	EAC tissue		peritumoral tissue	
			**high *IL10*** (n=19)	**low *IL10*** (n=20)	**high *IL10*** (n=15)	**low *IL10*** (n=16)
**age (years)**		61.9 (39.7 - 83.1)	62.6 (39.7 - 83.0)	61.3 (49.8-83.1)	62.2 (47.5 - 83.1)	63.9 (53.7 - 77.6)
**gender**	m	36 (92.3%)	16 (84.3%)	20 (100.0%)	14 (93.3%)	15 (93.8%)
	f	3 (7.7%)	3 (15.7%)	0 (0.0%)	1 (6.7%)	1 (6.2%)
**T**	1	6 (15.4%)	2 (10.5%)	4 (20.0%)	1 (6.7%)	5 (31.3%)
	2	2 (5.1%)	0 (0.0%)	2 (10.0%)	1 (6.7%)	0 (0.0%)
	3	24 (61.5%)	13 (68.4%)	11 (55.0%)	11 (73.3%)	9 (56.3%)
	4	7 (17.9%)	4 (21.1%)	3 (15.0%)	2 (13.3%)	2 (12.5%)
**N**	0	7 (17.9%)	5 (26.3%)	2 (10.0%)	2 (13.3%)	4 (25.0%)
	1	10 (25.6%)	7 (36.9%)	3 (15.0%)	3 (20.0%)	4 (25.0%)
	2	10 (25.6%)	2 (10.5%)	8 (40.0%)	7 46.7%)	2 (12.5%)
	3	12 (30.8%)	5 (26.3%)	7 (35.0%)	3 (20.0%)	6 (37.5%)
**M**	0	33 (84.6%)	17 (89.5%)	16 (80.0%)	12 (80.0%)	14 (87.5%)
	1	6 (15.4%)	2 (10.5%)	4 (20.0%)	3 (20.0%)	2 (12.5%)
**UICC**	1	4 (10.3%)	2 (10.5%)	2 (10.0%)	1 (6.7%)	3 (18.8%)
	2	6 (15.4%)	4 (21.1%)	2 (10.0%)	1 (6.7%)	3 (18.8%)
	3	23 (58.9%)	11 (57.9%)	12 (60.0%)	10 (66.7%)	8 (50.0%)
	4	6 (15.4%)	2 (10.5%)	4 (20.0%)	3 (20.0%)	2 (12.5%)
**L**	0	4 (10.3%)	2 (10.5%)	2 (10.0%)	1 (6.7%)	3 (18.8%)
	1	35 (89.7%)	17 (89.5%)	18 (90.0%)	14 (93.3%)	13 (81.3%)
**V**	0	23 (58.9%)	10 (52.6%)	13 (65.0%)	8 (53.3%)	12 (75.0%)
	1	16 (41.0%)	9 (47.4%)	7 (35.0%)	7 46.7%)	4 (25.0%)
**R**	0	27 (69,2%)	13 (68.4%)	14 (70.0%)	9 (60.0%)	11 (68.8%)
	1	12 (30.8%)	6 (31.6%)	6 (30.0%)	6 (30.0%)	5 (31.3%)
**G**	1	3 (7.7%)	1 (5.3%)	2 (10.0%)	1 (6.7%)	2 (12.5%)
	2	9 (23.1%)	6 (31.6%)	3 (15.0%)	4 (26.7%)	3 (18.8%)
	3	27 (69,2%)	12 (63.2%)	15 (75.0%)	10 (66.7%)	11 (68.8%)
**BMI (kg/m^2)**		29.2 (20.5 - 50.5)	27.2 (20.5 - 36.7)	31.1 (22.2 - 50.5)	29.9 (23.9 - 40.0)	28.9 (20.5 - 50.5)
**reflux**	yes	27 (69.2%)	13 (68.4%)	14 (70.0%)	10 (66.7%)	10 (62.5%)
	no	12 (30.8%)	6 (31.6%)	6 (30.0%)	5 (33.3%)	6 (37.5%)
**dead**	yes	28 (71.8%)	17 (89.5%)	11 (55.0%)	11 (73.3%)	11 (68.8%)
	no	11 (28.2%)	2 (10.5%)	9 (45.0%)	4 (26.7%)	5 (31.3%)
**survival (months)**		24.6 (2.3 - 77.9)	16.9 (2.3 - 44.2)	32.3 (5.0 – 77.9)	15.9 (2.3 - 51.7)	29.1 (4.0 - 49.5)

Patients are grouped into high and low by their median cutoff for relative *IL10* expression levels in EAC and peritumoral samples, respectively. Patients that died within 30 days after surgery (n=5) were excluded from survival data. Values are stated as mean ± range or % of group, respectively. EAC: esophageal adenocarcinoma; T: T

status (size and depth of tumor invasion); N: N status (tumor involvement in nearby lymph nodes); M status (distant metastasis); UICC (Union for International Cancer Control): comprising the TNM categories ; L: tumor invasion into lymphatic vessel; V: tumor invasion into vein; R: residual tumor at resection margin; G: grading of tumor cells; BMI: Body Mass Index

Table S2: Probes used in real-time PCR analysis.

Gene	Reference
IL22	Hs01574154_m1
IL22RA1	Hs00222035_m1
IL22RA2	Hs00364814_m1
IL17A	Hs00174383_m1
TNF	Hs01113624_g1
IFNG	Hs00989291_m1
IL10	Hs00961622_m1
FOXP3	Hs01085834_m1
CTLA4	Hs03044418_m1
PD1	Hs0155088_m1
HPRT1	Hs02800695_m1

All from Applied Biosystems.
